# RACIAL-ETHNIC DISPARITIES IN ATTITUDES TOWARD PASSIVE AND ACTIVE EUTHANASIA

**DOI:** 10.1093/geroni/igz038.1590

**Published:** 2019-11-08

**Authors:** Sara M Moorman

**Affiliations:** Boston College, Chestnut Hill, Massachusetts, United States

## Abstract

This paper examined racial/ethnic differences in opinions about passive euthanasia (withdrawing or withholding treatment), suicide, and physician-assisted suicide. Data came from 1,832 participants in the 2013 Pew Religion and Public Life Project. Respondents from all racial/ethnic backgrounds were most likely to favor multiple forms of euthanasia. However, persons of color had a wider variety of opinions about euthanasia than did non-Hispanic whites. In multivariate multinomial logistic regressions, non-Hispanic whites had a 63% chance of approving broadly of euthanasia, while non-Hispanic blacks had a 40% chance, and Hispanics, a 49% chance. Opposition to euthanasia was most common among people with multiple disadvantages (e.g., educational attainment, immigrant status). Neither trust in health care providers nor recent experience with the death of a loved one explained these group differences. Results highlight large differences of opinion between the people who set policy and practice guidelines and those who lack this power and access.

